# Health-related quality of life and caregiver burden of pediatric patients with inborn errors of metabolism in Japan using EQ-5D-Y, PedsQL, and J-ZBI

**DOI:** 10.1007/s11136-024-03775-0

**Published:** 2024-09-04

**Authors:** Keiko Konomura, Chikahiko Numakura, Akari Nakamura-Utsunomiya, Eri Hoshino, Go Tajima, Hironori Kobayashi, Kimitoshi Nakamura, Nobuyuki Shimozawa, Ryosuke Bo, Takeru Shiroiwa, Yosuke Shigematsu, Takashi Fukuda

**Affiliations:** 1 Center for Outcomes Research and Economic Evaluation for Health (C2H), National Institute of Public Health, 2-3-6 Minami, Wako-shi, Saitama, 351-0197 Japan; 2 Department of Clinical Genomics & Pediatrics, Saitama Medical University, 38 Moro Hongo Moroyama Iruma-Gun, Saitama, 350-0495 Japan; 3 Department of Pediatrics, Yamagata University School of Medicine, 2-2-2 Iida-nishi, Yamagata-shi, Yamagata, 990-9585 Japan; 4 Department of Pediatrics, Hiroshima University Graduate School of Biomedical and Health Sciences, 1-2-3 Kasumi, Minami-ku, Hiroshima-shi, Hiroshima, 734-8553 Japan; 5 Division of Policy Evaluation, Department of Health Policy, National Center for Child Health and Development, Research Institute, 2-10-1 Okura, Setagaya-ku, Tokyo, 157-8535 Japan; 6 Division of Neonatal Screening, National Center for Child Health and Development, Research Institute, 2-10-1 Okura, Setagaya-ku, Tokyo, 157-8535 Japan; 7 Laboratories Division, Shimane University Hospital, 89-1 Enya-cho, Izumo-shi, Shimane, 693-8501 Japan; 8 Department of Pediatrics, Graduate School of Medicine Sciences, Kumamoto University, 1-1-1 Honjo, Chuo-ku, Kumamoto-shi, Kumamoto, 860-8556 Japan; 9 Division of Genomics Research, Life Science Research Center, Gifu University, 1-1, Yanagido, Gifu-shi, Gifu, 501-1193 Japan; 10 Department of Pediatrics, Gifu University Graduate School of Medicine, Gifu University, 1-1, Yanagido, Gifu-shi, Gifu, 501-1193 Japan; 11 Clinical Genetics Center, Gifu University Hospital, 1-1 Yanagido, Gifu-shi, Gifu, 501-1194 Japan; 12 Department of Pediatrics, Kobe University Graduate School of Medicine, 7-5-1 Kusunoki-cho, Chuo-ku, Kobe, 650-0017 Japan; 13 Department of Pediatrics, University of Fukui School of Medical Sciences, Fukui, 910-1193 Japan

**Keywords:** Metabolism, Inborn errors, EQ-5D-Y, PedsQL, J-ZBI, Quality of Life

## Abstract

**Purpose:**

Inborn errors of metabolism (IEM) are known with poor long-term health concerns; however, the health-related quality of life (HRQoL) and the burden placed on families remain unclear. This study investigated the self- and proxy-reported HRQoL of pediatric patients with IEM with or without developmental disabilities and the burden placed on their caregivers.

**Methods:**

Patients with IEM aged 8–15 years and their caregivers were asked to respond to the Pediatric Quality of Life Inventory (PedsQL), EuroQoL five-dimension questionnaire for younger populations (EQ-5D-Y), and Japanese version of the Zarit Caregiver Burden Interview (J-ZBI). We compared EQ-5D-Y scores with matched EQ-5D-Y population norms. Intraclass correlation coefficients (ICC) for self and proxy HRQoL scores of those without developmental disabilities were calculated. Correlation coefficients of HRQoL proxy responses with J-ZBI score were estimated.

**Results:**

We included 66 patients with IEM (mean age, 11.5 years; males, 41.2%) in the study. The mean (± standard deviation) EQ-5D-Y scores without and with developmental disabilities were 0.957 (± 0.071) and 0.821 (± 0.175), respectively. The EQ-5D-Y scores significantly increased compared with the reference values (p < 0.01, effect size = 0.337). The ICC values were 0.331 and 0.477 for the EQ-5D-Y and PedsQL scores, respectively. HRQoL proxy scores had strong negative correlations with J-ZBI scores.

**Conclusion:**

The HRQoL of patients with IEM without developmental disabilities in our study was similar to that of the general Japanese population. The HRQoL of patients with IEM with developmental disabilities was low and associated with a tendency towards an increased burden of care.

## Plain English summary

Inborn errors of metabolism (IEM) are a large group of rare inherited disorders. Due to the long-term impact of IEM on childhood development, there are concerns about how this affects the patient’s health-related quality of life (HRQoL) and increases the care burden placed on their parents. In this study, we assessed the HRQoL of the pediatric patients with IEM and the burden placed on their families. This study indicated that the HRQoL of patients in our study without developmental disabilities was similar to that of the general population in Japan. Furthermore, patients with IEM with developmental disabilities tend to report lower HRQoL and place a higher burden on their caregivers. Our findings recommend further research to address the health policy challenges associated with patients with IEM.

## Introduction

Inborn errors of metabolism (IEM) are a large group of rare inherited disorders resulting from a defective metabolic pathway. IEM are associated with poor long-term health outcomes, including metabolic, developmental, and intellectual disabilities, as well as increased mortality [1]. Since early intervention can prevent the negative health impact of some IEM diseases, newborn screening (NBS) for IEM is conducted in each country [2], and this has increased the rate of detection of IEM.

Due to the long-term impact of IEM on childhood development, there are concerns about how this affects the patient’s health-related quality of life (HRQoL) and increases the care burden placed on their parents [3]. The long-term necessity of compliance with strict diet therapy and the occurrences of cognitive symptoms can affect behavioral and psychosocial aspects of HRQoL [4, 5]. Several studies have reported the HRQoL for relatively common conditions, such as phenylketonuria (PKU) and maple syrup urine disease (MSUD) [5–7]. However, for several IEMs, the HRQoL of the patients and the burden placed on their families remain unclear. Furthermore, the extent of the decrease in HRQoL due to the occurrence of developmental disorders, an important concern in patients with IEM, has not yet been reported. Additionally, the use of generic preference-based measures of HRQoL in patients with IEM has not yet been reported. These measures provide a common index that enables comparisons across different areas of health interventions [8].

HRQoL measurement has become particularly important in recent years owing to the growing interest in rare diseases and the potential need for health economic evaluation. The HRQoL measurement in pediatric patients should be assessed by themselves [9]. However, self-repoted HRQoL assessment may not be feasible for personal characteristics such as developmental level and cognitive impairment. Therefore, proxy assessments of HRQoL are commonly performed by parents and health professionals. The previous study reported poor inter-rater agreement for overall HRQoL between proxy and self-respondents [10, 11], and various potential factors, such as health conditions, affect the direction and magnitude of the differences [12]. Therefore, it is important to collect quantitative information to understand the properties of proxy responses for patients with IEM.

Measuring HRQoL can help health care providers and policymakers to understand how IEM affects both the patients and their families and to provide them with information to implement necessary interventions. Hence, this observational study of pediatric patients with IEM used the results of self- and proxy-reported HRQoL questionnaires as well as the Japanese version of the Zarit Caregiver Burden Interview (J-ZBI) in an effort to contribute to the knowledge on the HRQoL of pediatric patients with IEM and the burden placed on their caregivers in Japan, particularly for patients with IEM and developmental disabilities.

## Methods

### Participants and study design

This noninterventional, observational, multicenter study was conducted in Japan from October 2019 to March 2021. Due to the lack of a comprehensive way to identify IEM patients, the study participants were recruited from 34 hospitals where IEM specialists are assigned. Patients with IEM aged 8–15 years and their caregivers were included in this study. Caregivers were defined as parents or any persons with parental authority. Patients with the following 22 IEM disorders were included: six amino acid disorders, eight organic acid disorders, and eight fatty acid oxidation disorders (Table 1). If the patients had difficulty in answering, only their caregivers participated in the study. Participation was optional and informed consent was obtained from all participants. This study was approved by the Ethical Review Committee of Yamagata University Faculty of Medicine (#2019-227) and registered in the UMIN Clinical Trials Registry (UMIN000038674).

**Table 1:** The list of targeted disease

Amino acid disorders
Phenylketonuria
Maple syrup urine disease
Homocystinuria type 1
Citrullinemia type 1
Argininosuccinic aciduria
Citrin deficiency
Organic acid disorders
Methylmalonic acidemia
Propionic acidemia
Isovaleric acidemia
Methylcrotonylglycinuria
3-hydroxy-3-methylglutaric acidemia
Multiple carboxylase deficiency
Glutaric acidemia type 1
Beta-ketothiolase deficiency
Fatty acid oxidation disorders
Primary carnitine deficiency (caused by organic cation transporter 2 deficiency)
Carnitine palmitoyltransferase I deficiency
Carnitine-acylcarnitine translocase deficiency
Carnitine palmitoyltransferase II deficiency
Very-long-chain acyl-CoA dehydrogenase deficiency
Mitochondrial trifunctional protein deficiency
Medium-chain acyl-CoA dehydrogenase deficiency
Glutaric acidemia type 2

### Procedure

The details of the study were explained to the patients who met the eligibility criteria and their caregivers by the attending physician, and all participants provided informed consent. The attending physician reviewed the participants’ medical charts, and their background information was recorded at the data center. Participants responded anonymously to the questionnaire via mail. The data center integrated the questionnaire responses according to the patient number assigned by the patient registry.

### Measures

The following background information was collected for each patient: age, sex, confirmed diagnosis, detection by NBS, dietary management, physical growth, developmental status, severity of the developmental disabilities, and caregivers’ age, sex and relationship. The severity of developmental disabilities was classified by the patient’s attending physician as mild, moderate, or severe. Mild developmental disability was defined as developmental delay in patients who could perform daily activities normally. Moderate developmental disability was defined as requiring assistance to perform daily activities. Severe developmental disability was defined as difficulty communicating or walking independently. Patients were asked to complete two validated Japanese versions of HRQoL assessment questionnaires: the EuroQoL five-dimension questionnaire for younger populations (EQ-5D-Y) Japanese version and the Pediatric Quality of Life Inventory, version 4.0 (PedsQL) [13, 14]. The EQ-5D-Y measures five dimensions of pediatric HRQoL (mobility; looking after myself; doing usual activities; having pain or discomfort; and feeling worried, sad, or unhappy) [13–20]. Each dimension comprised three levels: “no problems,” “some problems,” and “a lot of problems”. The patient’s health state profile can be represented by combining the digits of five dimensions; for instance, “11111” represents full health and “33333” represents the worst health state. A single summary score for the health status, often used in economic evaluation, can derive converted from the health profile using validated value sets. We used the Japanese value set, which has values ranging from 1 for a full health state to 0.289 for the worst health state [21]. The PedsQL measures 23 items, resulting in a total HRQoL score and two summary scores: physical health and psychosocial health [15–20]. The physical health score comprises the physical health functioning domain score, and the psychosocial health score comprises the emotional, social, and school functioning domain scores. Each item is recorded on a five-point Likert scale, and the scores are then transformed and integrated into a scale ranging from 0 to 100, with lower values indicating a lower HRQoL.

Caregivers were asked to complete the proxy measures for the EQ-5D-Y (proxy version 1) and PedsQL [13–20]. In addition, they were also asked to respond to the J-ZBI questionnaire [22, 23]. The J-ZBI measures the total burden of caregiving, including the physical burden, psychological burden, and financial difficulties. It consists of 22 questions about the comprehensive burden of care, and the responses are recorded on a 5-point Likert scale. The J-ZBI score ranges from 0 to 88 points. The higher the J-ZBI score, the heavier the burden of care.

### Data analysis

Internal consistency of total and subscale scores for PedsQL and J-ZBI was assessed using Mc Donald’s coefficients (total omega, and omega hierarchical) [24, 25]. The HRQoL responses were analyzed using descriptive statistics, and these were classified according to developmental diagnosis and disease. Due to the small number of patients, we combined some of the disease categories for aggregation. We compared our EQ-5D-Y scores with reference values that matched the patients’ age and sex from published EQ-5D-Y population norms for Japanese children [26]. We used the Wilcoxon rank-sum test to compare group differences between the EQ-5D-Y scores and reference values. Statistical significance was set at p < 0.05. A one-way random-effects model was used to calculate the intraclass correlation coefficients (ICC) for the HRQoL scores to evaluate the inter-rater agreement between the self-completed and proxy responses. ICC values less than 0.5, between 0.5 and 0.75, between 0.75 and 0.9, and greater than 0.9 indicate poor, moderate, good, and excellent agreement, respectively [27]. Correlations between HRQoL proxy responses and J-ZBI scores were calculated using Spearman’s rank correlation coefficients. Only HRQoL proxy responses were used because patients with developmental disabilities are expected to have difficulty responding in person. Descriptive statistics were computed with the SAS version 9.4 (SAS Institute Inc., Cary, NC, USA), while the estimation of internal consistency was conducted with the R package Psych.

## Results

### Patients

A total of 67 patients were enrolled between from October 2019 and March 2021. One patient was excluded from the study after responding neither to self- nor proxy-HRQoL questionnaires; therefore, 66 patients were finally included in the study. Of these, 55 completed self-reports for each HRQoL questionnaire. All caregivers (N = 66) provided valid responses to at least one HRQoL questionnaire. The number of caregivers who completed the EQ-5D-Y, PedsQL, and J-ZBI questionnaires was 64, 66, and 63, respectively. The demographic and clinical characteristics of the patients are presented in Table 2. The mean (standard deviation [SD]) age of the patients was 11.5 (2.1) years and 28 patients (42.4%) were male. A total of 35 patients (53.0%) were found through NBS. The most prevalent IEM in our study cohort was PKU (24 patients, 36.4%), followed by citrin deficiency (nine patients, 13.6%) and MSUD (six patients, 9.1%). There were 48 (72.7%) patients with no developmental disabilities, three (4.5%) with borderline disabilities, and 15 (22.7%) with developmental disabilities; seven (46.7%), six (40.0%), and two (13.3%) patients had mild, moderate, and severe developmental disabilities, respectively.

**Table 2:** Patient characteristics (N = 66)

Mean age in years (standard deviation [SD])	11.5	(2.14)
Sex, n (%)		
Missing	1	(1.52)
Female	37	(56.06)
Male	28	(42.42)
Diagnosis, n (%)		
Phenylketonuria	24	(36.36)
Citrin deficiency	9	(13.64)
Maple syrup urine disease	6	(9.09)
Methylmalonic acidemia	5	(7.58)
Propionic acidemia	4	(6.06)
Citrullinemia type 1	3	(4.55)
Methylcrotonylglycinuria	2	(3.03)
Glutaric acidemia type 1	2	(3.03)
Very-long-chain acyl-CoA dehydrogenase deficiency	2	(3.03)
Carnitine palmitoyltransferase I deficiency	2	(3.03)
Carnitine palmitoyltransferase II deficiency	2	(3.03)
Glutaric acidemia type 2	2	(3.03)
Others	3	(4.54)
Diagnosed using NBS, n (%)	35	(53.03)
Dietary management, n (%)		
No dietary restrictions	13	(19.70)
Dietary restrictions	50	(75.75)
Tube feeding	3	(4.55)
Physical growth n, (%)		
Normal range	55	(83.32)
Short stature	7	(10.61)
Underweight	3	(4.55)
Overweight	1	(1.52)
Developmental status, n (%)		
Normal range	48	(72.72)
Borderline	3	(4.55)
Had developmental disabilities	15	(22.73)
Severity of developmental disabilities n, (%)		
Mild	7	(46.67)
Moderate	6	(40.00)
Severe	2	(13.33)
All caregivers, n (%)	66	(100)
Mean age in years (SD)	42.5	(5.18)
Sex, n (%)		
Missing	6	(9.09)
Male	4	(6.06)
Female	56	(84.85)
Relationship, n (%)		
Missing	18	(27.27)
Father	2	(3.03)
Mother	46	(69.70)

Mild developmental disabilities: developmental delay but the ability to perform daily activities normally, moderate developmental disabilities: requires assistance in performing daily activities, severe developmental disabilities: difficulty communicating or walking independently

### Reliability

Reliability estimates (omega-t and omega-h) of the PedsQL overall scores were 0.95 and 0.58 for the self-responses, and 0.98 and 0.78 for the proxy-responses. Omega-t of the PedsQL subdomains as physical, emotional, social, and school functioning were 0.90, 0.88, 0.95, and 0.95 in the self-responses and 0.97, 0.96, 0.95, and 0.95 in the proxy-responses. Omega-t of the total scores for the J-ZBI was 0.96.

### Health-related quality of life

The EQ-5D-Y self- and proxy-health states collected from patients with IEM had 12 and 19 unique combinations, respectively (Table 3). The most commonly responded health states were “11111” (63.6%) and “11112” (14.5%) for the self-health state and “11111” (62.5%) and “11112” (7.8%) for the proxy-health states. The worst responded health state was “22233” (1.8%) in the self-health state and “33333” (1.6%) in the proxy-health state.

**Table 3:** Distribution of health state from EQ-5D-Y

Health state	EQ-5D-Y (n = 55)		EQ-5D-Y proxy (n = 64)	
	n	%	n	%
11111	35	(63.64)	40	(62.5)
11112	8	(14.55)	5	(7.81)
11113	1	(1.82)	1	(1.56)
11121	1	(1.82)	0	(0)
11122	1	(1.82)	0	(0)
11211	0	(0)	1	(1.56)
11212	0	(0)	1	(1.56)
11221	1	(1.82)	1	(1.56)
11222	0	(0)	2	(3.13)
12111	1	(1.82)	2	(3.13)
12112	0	(0)	1	(1.56)
12122	1	(1.82)	0	(0)
12211	0	(0)	1	(1.56)
12322	1	(1.82)	0	(0)
13111	0	(0)	1	(1.56)
21111	2	(3.64)	1	(1.56)
21112	0	(0)	1	(1.56)
21222	2	(3.64)	1	(1.56)
22112	0	(0)	1	(1.56)
22233	1	(1.82)	0	(0)
23211	0	(0)	1	(1.56)
23322	0	(0)	1	(1.56)
32211	0	(0)	1	(1.56)
33333	0	(0)	1	(1.56)

Table 4 shows the HRQoL scores of the patients with IEM without developmental disabilities. The mean (SD) reference value for the general population matched for age and sex of patients with IEM without developmental disabilities who responded to the EQ-5D-Y in person was 0.932 (0.006). The EQ-5D-Y scores of patients with IEM without developmental disabilities significantly increased compared with the reference values (p < 0.001, effect size = 0.337). The mean EQ-5D-Y and EQ-5D-Y proxy scores of patients with IEM without developmental disabilities ranged from 0.927 to 1.000 and from 0.950 to 1.000, respectively. The mean scores of PedsQL and PedsQL proxy of patients with IEM without developmental disabilities ranged from 88.0 to 96.7 and from 87.0 to 95.8, respectively. Table 5 shows the HRQoL scores of the patients with IEM with developmental disabilities, among whom most responses were proxy responses. The mean HRQoL scores tended to be lower in patients with developmental disabilities than that in patients without developmental disabilities. The citrullinemia type 1 group, which had the lowest HRQoL proxy-response, included patients diagnosed with severe degree of developmental disabilities.

**Table 4:** HRQoL scores of patients with IEM without developmental disabilities

Diseases	EQ-5D-Y			EQ-5D-Y proxy		
	n	Mean	SD	n	Mean	SD
All patients	50	0.957	0.071	50	0.979	0.047
Amino acid disorders	26	0.962	0.073	26	0.991	0.024
Phenylketonuria*	23	0.957	0.077	22	0.990	0.026
Maple syrup urine disease	3	1.000	0.000	3	1.000	0.000
Urea cycle disorders (citrin deficiency)	9	0.927	0.095	9	0.950	0.082
Organic acid disorders	7	0.955	0.044	7	0.976	0.041
Methylmalonic acidemia	2	0.949	0.072	2	0.963	0.052
Propionic acidemia	2	0.931	0.006	2	0.954	0.065
Fatty acid oxidation disorders	8	0.973	0.053	8	0.974	0.052

	PedsQL			PedsQL proxy		
	n	Mean	SD	n	Mean	SD
All patients*	50	93.6	8.0	51	93.5	8.3
Amino acid disorders*	26	93.6	8.2	27	95.7	5.3
Phenylketonuria	23	93.5	8.5	23	95.8	5.0
Maple syrup urine disease	3	94.6	6.1	3	93.5	7.8
Urea cycle disorders (citrin deficiency)	9	90.0	11.2	9	89.6	13.5
Organic acid disorders	7	94.9	5.0	7	93.5	6.6
Methylmalonic acidemia	2	96.7	3.1	2	87.0	1.5
Propionic acidemia	2	88.0	1.5	2	91.3	7.7
Fatty acid oxidation disorders	8	96.3	3.8	8	90.8	9.9

*SD* standard deviation

*Missing data are shown

**Table 5:** HRQoL scores of patients with IEM with developmental disabilities

Disease	EQ-5D-Y			EQ-5D-Y proxy			PedsQL			PedsQL proxy		
	n	Mean	SD	n	Mean	SD	n	Mean	SD	n	Mean	SD
All patients	5	0.878	0.239	14	0.821	0.175	5	73.8	19.8	15	55.8	19.5
Amino acid metabolism disorders	3	0.796	0.299	7	0.781	0.223	3	64.9	21.3	7	51.6	26.0
Maple syrup urine disease	2	0.727	0.387	3	0.859	0.083	2	63.6	30.0	3	61.6	8.3
Citrullinemia type 1	–	–	–	2	0.589	0.424	–	–	–	2	38.0	53.8
Organic acid disorders	–	–	–	6	0.867	0.122	–	–	–	7	60.7	12.6
Methylmalonic acidaemia	–	–	–	3	0.795	0.143	–	–	–	3	53.6	9.7
Propionic acidaemia	–	–	–	2	0.931	0.037	–	–	–	2	69.6	9.2

Responses from the following disease groups with only one respondent are not shown in the table for ethical reasons: self-report of organic acid disorder and self- and proxy-report of fatty acid oxidation disorder. Specific diseases with only one respondent within the disease groups were not shown for the same reason

*SD* standard deviation

**Fig. 1:**
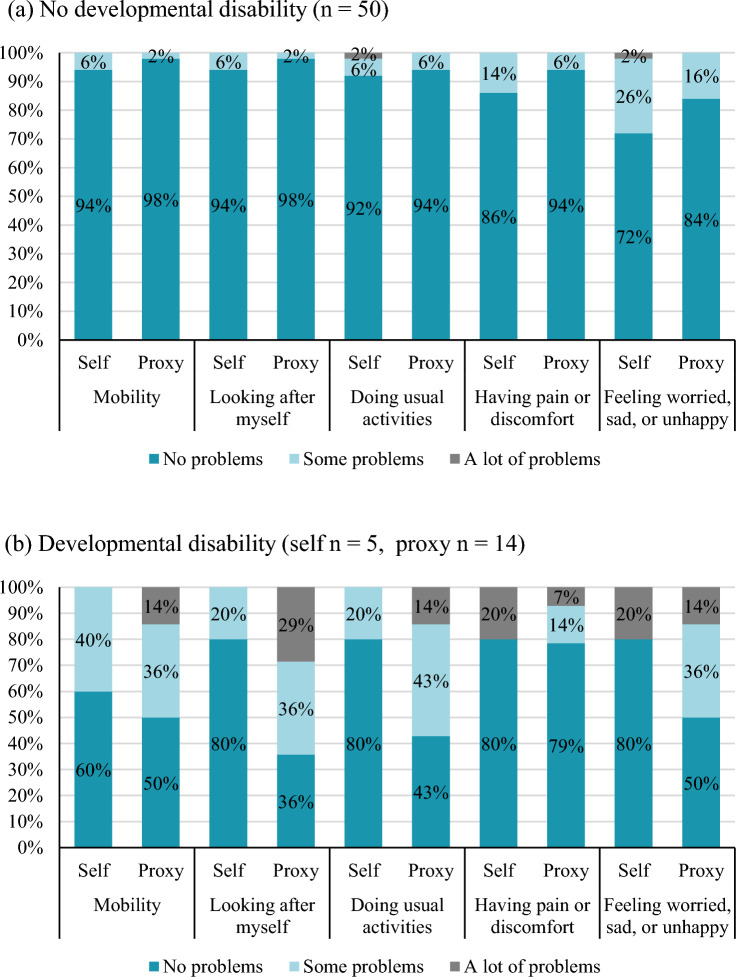
Distribution of responses in the domains of EQ-5D-Y

The ICC values for the correlation between the self- and proxy-reported HRQoL scores of patients with IEM without developmental disabilities on the EQ-5D-Y and PedsQL were 0.331 and 0.477, respectively. 72% and 84% of the self- and proxy-respondents of patients with IEM without developmental disabilities reported “no problems” in the “feeling worried, sad, or unhappy” domain (Fig. 1). In the proxy responses for patients with IEM with developmental disabilities, over 50% of the respondents answered that they had problems, except for the “having pain or discomfort” domain. Table 6 shows the distribution of domain responses to the PedsQL questionnaire. The mean (SD) emotional functioning domains of the self- and proxy-respondents of patients with IEM without developmental disabilities were 92.3 (12.9) and 94.8 (9.6). The mean (SD) school functioning domains of the self- and proxy-respondents of patients with IEM without developmental disabilities were 92.1 (9.4) and 90.6 (12.1). Most proxy respondents of patients with IEM with developmental disabilities reported that all domains problematic, and the mean (SD) physical, emotional, social, and school functioning domains were 53.1 (28.6), 68.7 (24.5), 54.0 (20.0), and 49.0 (24.2), respectively.

**Table 6:** Scale descriptives for PedsQL 4.0 Generic Core Scales self- and proxy-reported results without or with developmental disability

	Self						Proxy					
	n	Mean	SD	Median	25%tile	75%tile	n	Mean	SD	Median	25%tile	75%tile
Without developmental disability												
Physical functioning	50	93.8	9.2	90.6	100.0	100.0	51	93.9	10.7	100.0	90.6	100.0
Emotional functioning	50	92.3	12.9	90.0	100.0	100.0	51	94.8	9.6	100.0	95.0	100.0
Social functioning	50	95.9	9.1	95.0	100.0	100.0	51	94.7	12.5	100.0	100.0	100.0
School functioning	50	92.1	9.4	85.0	100.0	100.0	51	90.6	12.1	95.0	85.0	100.0
With developmental disability												
Physical functioning	5	76.6	23.5	89.3	56.3	90.6	15	53.1	28.6	56.3	25.0	75.0
Emotional functioning	5	73.0	29.7	75.0	70.0	95.0	15	68.7	24.5	70.0	55.0	85.0
Social functioning	5	74.8	28.3	85.0	65.0	93.8	15	54.0	20.0	60.0	45.0	70.0
School functioning	5	70.0	8.7	65.0	65.0	70.0	15	49.0	24.2	55.0	35.0	70.0

*SD* standard deviation

### Caregiver burden and correlation of HRQoL proxy scores

The mean (SD) J-ZBI scores for caregivers of patients with IEM without and with developmental disabilities were 7.5 (5.9) and 27.3 (16.8), respectively. The mean (SD) J-ZBI scores for caregivers of IEM patients with mild, moderate, and severe developmental disabilities were 23.7 (8.2), 21.6 (11.3), and 54.5 (31.8), respectively. Comparing the HRQoL proxy scores and the J-ZBI scores, the EQ-5D-Y proxy scores were negatively correlated with J-ZBI scores (Spearman's rank correlation =  − 0.798, p < 0.001) and the PedsQL proxy scores (− 0.578, p < 0.001).

## Discussion

We measured the HRQoL of patients with IEM using the EQ-5D-Y and PedsQL questionnaires. In our study, the EQ-5D-Y scores of patients with IEM without developmental disabilities were higher than the reference values for the general population of Japanese children. In addition, 64% of the patients with IEM reported perfect health. Although reference values for PedsQL are not available in Japan, Okano et al. reported the mean (SD) PedsQL value in the healthy child sample in Japan was 80.9 (12.4) [28]. These results indicate that the patients with IEM without developmental disabilities in our study demonstrated minor health problems and achieved an HRQoL comparable with that of the general population. Several previous studies on PKU have been reported, and those studies that used general questionnaire have showed that patients with PKU have HRQoL scores comparable to or better than healthy controls [29–31]. In a European multinational study of patients with PKU that was conducted using the PedsQL, the total scale scores for children and adolescents were 85.5 and 85.1, respectively, which is in line with our study [30]. In Japan, newborns have been screened for PKU since 1977, which is covered by the universal health insurance system. Therefore, it is likely that patients with PKU can receive continuous medical services after early detection of the disease. However, previous studies have not sufficiently reported the severity of the disease or developmental disabilities; therefore, a detailed comparison of the results could not be made. The results of this study suggest that patients with IEM but without developmental disabilities may achieve an HRQoL comparable to that of the general population.

To the best of our knowledge, this is the first study to report the HRQoL in patients with IEM and developmental disabilities. A previous study reported that the EQ-5D-Y score for patients with developmental disabilities was approximately 0.85 [26]. Notably, the scores in our study were similar to or lower than previously reported values. The previous study was conducted in a community population and did not include children and adolescents with severe diseases. Conversely, our study included patients with IEM with severe developmental disabilities, and the measured quantitative HRQoL value in these patients will be an important reference for future health economic evaluations.

The ICC values were < 0.5 for both the EQ-5D-Y and PedsQL, and the agreements between patients with IEM without developmental disabilities and their proxy responses was poor. The proxy HRQoL responses tended to score higher than the self-completed HRQoL responses in psychological domains. Previous studies have similarly reported differences in the agreement of overall scores, especially psychosocial-related domains between self- and proxy responses [10, 32].

In our study, self-responses of patients with IEM with mild developmental disabilities were also reported. However, the interpretation of the results, especially the differences between the self- and proxy-respondents in Tables 5 and 6, should be noted as they might not reflect the actual differences because of the small sample size (n = 5), significant deviation and inconsistency from the paired proxy response, and the inability to confirm the patients' ability to self-assess their own. For the same reason, we excluded them from the analysis to estimate the ICC values. Further research is needed to quantify the HRQoL according to the type and degree of developmental disability.

The J-ZBI score tended to increase for caregivers of patients with IEM with developmental disabilities and was negatively correlated with the HRQoL proxy scores. The J-ZBI was developed for caregivers of older adults and is the only available questionnaire validated as a Japanese version to assess caregiver burden [23]. However, this has not been adequately validated for caregivers of children. Toki et al. conducted a survey of parents of children with disabilities and reported that groups with high (35.2) and low (14.5) J-ZBI scores showed 40.5% and 9.3% depressive states, respectively [33]. Several studies have been conducted on pre-translated versions of the Zarit Burden Interview in children [34–38]. However, it is unclear whether the degree of burden on caregivers can be interpreted in the same manner as in the pre-translated version. Our study demonstrated that low patient health status was associated with increased caregiver burden and showed the possibility of using the J-ZBI to assess child caregiver burden. Measuring caregiver burden for child caregivers is an important issue that requires further research.

This study had some limitations. First, there was a possible selection bias because the respondents were asked by their attending physicians to participate in the survey and respond by mail. As it is challenging to identify patients with rare IEM, sampling by specialists is considered the most effective method of data collection. Secondly, it was difficult to design a survey to ensure a sufficient sample size for each disease. We grouped IEM diseases with small sample sizes based on disease mechanisms. Therefore, detailed information on the HRQoL for each disease was lost. However, differences in the distribution of HRQoL according to disease were not evident among patients. Lastly, because our study was conducted via mail, there may be a possibility that the patient’s caregiver responded to the self-response on behalf of the patient. Although our results should be interpreted with caution, there are several important strengths, such as the quantification of HRQoL values of patients with IEM with or without developmental disabilities, analysis of both self- and proxy-reported measures of HRQoL, and measurement of HRQoL using generic preference-based measures in IEM patients.

## Conclusion

This study provides evidence of the HRQoL of patients with IEM. Our findings suggest that the HRQoL of patients with IEM without developmental disabilities in our study was similar to that of the general Japanese population. Furthermore, patients with IEM with developmental disabilities tend to have a lower HRQoL and place a higher burden on their caregivers. In recent years, the development of new drugs to treat rare diseases has emphasized the importance of NBS and the need for further research and health economic evaluations showing that NBS for IEM has a long-term positive economic effect.

## Data Availability

The datasets generated during the current study are not publicly available due to the lack of consent from participants, but are available from the corresponding author upon reasonable request.
